# Loss of Angiopoietin-like 7 diminishes the regeneration capacity of hematopoietic stem and progenitor cells

**DOI:** 10.1186/s13045-014-0102-4

**Published:** 2015-02-06

**Authors:** Yiren Xiao, Xinru Wei, Zhiwu Jiang, Xiangmeng Wang, Wei Ye, Xin Liu, Minjie Zhang, Yan Xu, Donghai Wu, Liangxue Lai, Huihui Yao, Zixia Liu, Su Cao, Pentao Liu, Bing Xu, Yangqiu Li, Yao Yao, Duanqing Pei, Peng Li

**Affiliations:** Key Laboratory of Regenerative Biology, South China Institute for Stem Cell Biology and Regenerative Medicine, Guangzhou Institutes of Biomedicine and Health, Chinese Academy of Sciences, 190 Kaiyuan Avenue, Science Park, Guangzhou, 7, Guangdong 510530 China; Guangdong Provincial Key Laboratory of Stem Cell and Regenerative Medicine, South China Institute for Stem Cell Biology and Regenerative Medicine, Guangzhou Institutes of Biomedicine and Health, Chinese Academy of Sciences, Guangzhou, 510530 China; Department of Hematology, Nanfang Hospital, Southern Medical University, 510515 Guangzhou, China; Shenzhen Institutes of Advanced Technology, Chinese Academy of Sciences, 1068 Xueyuan Avenue, Shenzhen University Town, Shenzhen, 518055 China; Institute of Hematology, Medical College, Jinan University, Guangzhou, 510632 China; Key Laboratory for Regenerative Medicine of Ministry of Education, Jinan University, Guangzhou, 510632 China; Department of Outpatient, The 91th Military Hospital, 454003 Jiaozuo, China; Division of Reproductive Endocrinology, The 91th Military Hospital, 454003 Jiaozuo, China; Division of General Pediatrics, The 91th Military Hospital, 454003 Jiaozuo, China; Wellcome Trust Sanger Institute, Hinxton, Cambridge CB10 1HH, England, UK; Drug Discovery Pipeline, Guangzhou Institutes of Biomedicine and Health, Chinese Academy of Sciences, Guangzhou, 510530 China

**Keywords:** Angptl7, Knockout-mice, Hematopoietic stem cell, Repopulation, Homing

## Abstract

Successful expansion of hematopoietic stem cells (HSCs) would benefit the use of HSC transplants in the clinic. Angiopoietin-like 7 promotes the expansion of hematopoietic stem and progenitor cells (HSPC) in vitro and ex vivo. However, the impact of loss of Angptl7 on HSPCs in vivo has not been characterized. Here, we generated *Angptl7*-deficient mice by TALEN-mediated gene targeting and found that HSC compartments in *Angptl7*-null mice were compromised. In addition, wild type (WT) HSPCs failed to repopulate in the BM of *Angptl7*-null mice after serial transplantations while the engraftment of *Angptl7*-deficient HSPCs in WT mice was not impaired. These results suggest that Angptl7 is required for HSPCs repopulation in a non-cell autonomous manner.

**Electronic supplementary material:**

The online version of this article (doi:10.1186/s13045-014-0102-4) contains supplementary material, which is available to authorized users.

## Findings

The autologous-allogeneic hematopoietic cell transplantation has been developed for decades [1,2], and numerous attempts have been made to expand the HSCs population *in vitro* and *in vivo* [3-5]. Agiopoietin-like proteins belong to a 7-member family of secreted glycoproteins that share sequence homology with angiopoietins, which are important modulators of angiogenesis [6]. Several Angptl family proteins promote the expansion of murine and human HSPCs in vitro and *ex vivo* [7-9]. Angptl7 in the ECM of the trabecular meshwork plays an important role in the deposition and organization of the matrix of the outflow tissue [10]. Recently, we found that ANGPTL7 stimulated the proliferation of human HSPCs ex vivo (Yiren Xiao, unpublished data). In addition, we uncovered that Angptl7, which was secreted by murine bone marrow SSEA4+ mesenchymal cells (Additional file 1: Figure S1), stimulated expansion of murine HSCs ex vivo (Additional file 2: Figure S2). However, whether Angptl7 is redundant and dispensable or not for repopulation of HSPCs in vivo remains unknown. In this study, we generated Angptl7 knockout mice and revealed that Angptl7 is essential for HSPC repopulation in a non-cell autonomous way.

**Figure 1:**
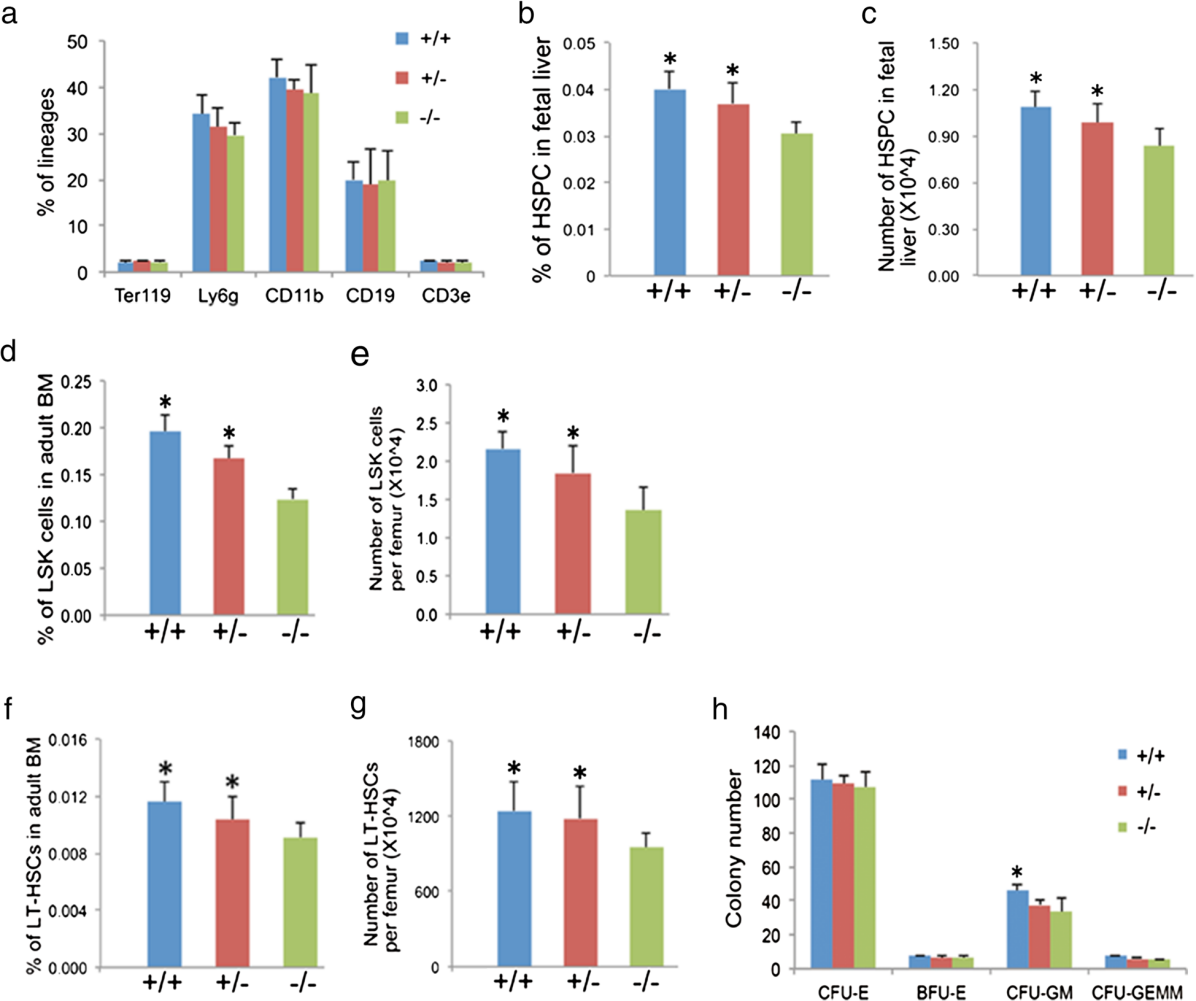
**Reduced HSC in Angptl7** ^**−/−**^ **mice. (a)** Characterization of multiple hematopoietic lineages in BM. The major lineages of hematopoietic cells in BM in *Angptl7* ^*−/−*^ *Angptl7* ^*+/−*^ and *Angptl7* ^*+/+*^ mice were quantitated by flow cytometry analysis as erythroid cells (Ter119), myeloid cells (Ly6g and CD11b), T cells (CD3e) and B cells (CD19). Data represent the means +/− s.e.m (*n* = 5 for each group). **(b, c)** Comparison of the frequencies **(b)** and number **(c)** of Lin-Mac-1 + CD4- population in the fetal liver of *Angptl7* ^*−/−*^ *Angptl7* ^*+/−*^ and *Angptl7* ^*+/+*^ mice. Data represent the means +/− s.e.m (*n* = 10 for each group). *P < 0.05 versus *Angptl7* ^*−/−*^ group for *Angptl7* ^*+/−*^ and *Angptl7* ^*+/+*^. **(d, e)** Comparison of the frequencies **(d)** and number **(e)** of LSK population in the BM of *Angptl7* ^*−/−*^ *Angptl7* ^*+/−*^ and *Angptl7* ^*+/+*^ mice. Data represent the means +/− s.e.m (*n* = 6 for each group). *P < 0.05 versus *Angptl7* ^*−/−*^ group for *Angptl7* ^*+/−*^ and *Angptl7* ^*+/+*^ group. **(f, g)** Comparison of the frequencies (left) and number (right) of LT-HSCs in the BM of *Angptl7* ^*−/−*^ *Angptl7* ^*+/−*^ and *Angptl7* ^*+/+*^ mice. Data represent the means +/− s.e.m (*n* = 5 for each group). *P < 0.05 versus *Angptl7* ^*−/−*^ group for *Angptl7* ^*+/−*^ and *Angptl7* ^*+/+*^ group. **(h)** The colony numbers of CFU assays obtained from BM of *Angptl7* ^*−/−*^ *Angptl7* ^*+/−*^ and *Angptl7* ^*+/+*^ mice. Data represent the means +/− s.e.m (*n* = 3). *P < 0.05 versus bar 7 for bar 9.

To investigate whether the loss of Angptl7 affected HSPCs *in vivo*, we generated Angptl7-null mice by TALEN-mediated gene targeting (Additional file 1: Figure S1a-1c). The procedures of gene targeting were described in (Additional file 3: Supplementary methods) and (Additional file 4: Table S1). Loss of Angptl7 in *Angptl7*-null mice was confirmed in the bone marrow (BM) by RT-PCR and Western blotting (Additional file 5: Figure S3d-3e). The *Angptl7*-null mice did not display an overt phenotype. There were no significant differences in either birth weights or adult weights among *Angptl7*^−/−^, *Angptl7*^+/−^, and *Angptl7*^+/+^ mice (Additional file 6: Table S2). In addition, we found no significant differences in staining profiles T cells, B cells, myeloid cells, and erythroid cells among the *Angptl7*^−/−^, *Angptl7*^+/−^ and *Angptl7*^+/+^ mice (Figure 1a).

As HSCs undergo dramatic expansion in fetal liver during embryo development [11-13], we examined the fetal liver HSC compartment and found that the percentages and numbers of Lin-Mac-1 + CD4- population, which was highly enriched of HSCs in fetal liver [14,15], were significantly lower in the fetal livers of *Angptl7*^−/−^ mice than that in *Angptl7*^+/−^ and *Angptl7*^+/+^ mice (Figure 1b-c). In addition, we found the percentages and numbers of Lin-Sca-1 + c-Kit + (LSK) population in the BM of adult *Angptl7*^−/−^ mice were significantly lower than that in *Angptl7*^+/−^ and *Angptl7*^+/+^ mice (Figure 1d-e). Further analysis showed that *Angptl7*^−/−^ mice had fewer long-term HSCs (Lin^−^Sca-1^+^Kit^+^Flk2^−^CD34^−^) than Angptl7^+/−^ and *Angptl7*^+/+^ mice (Figure 1f-g). Cloning forming assay results showed that BM cells from *Angptl7*^−/−^ mice had fewer granulocyte/monocyte progenitors (CFU-GM), but similar numbers of erythroid precursors (CFU-E/BFU-E) and hematopoietic progenitors (CFU-GEMM) than WT BM (Figure 1h). Therefore, *Angptl7*-deficient BM had normal levels of terminally differentiated hematopoietic cells, but decreased myeloid progenitors. To investigate whether other angiopoietin-like proteins compensate the loss of Angptl7 in BM, we compared the expression levels of other angiopoietin-like proteins in *Angptl7*-deficient BM stromal cells to that in WT BM stromal cells and found that their expression levels did not significantly change due to loss of Angptl7 (Additional file 7: Figure S4).

**Figure 2:**
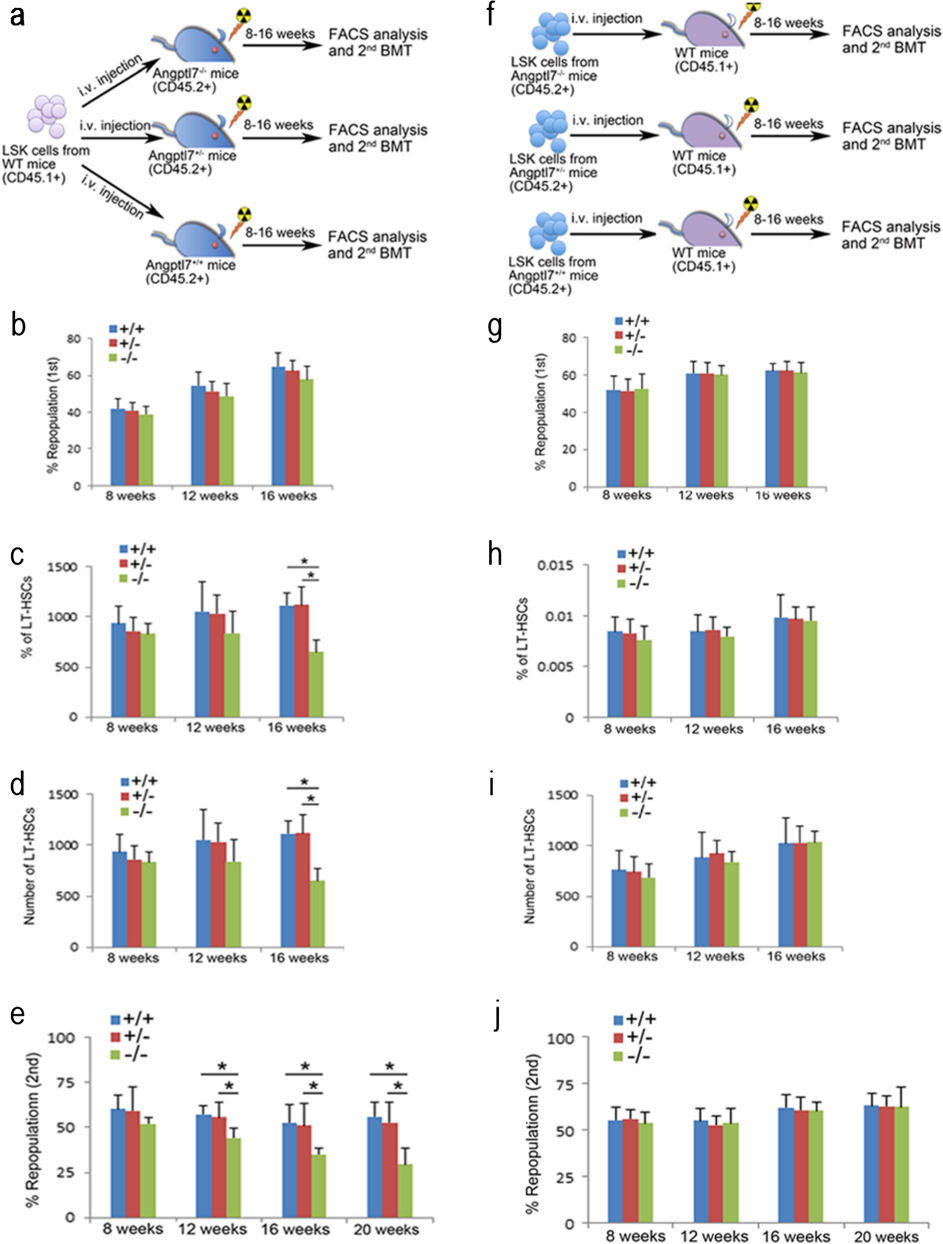
**Comparison of reconstitution capacity of HSC in Angptl7** ^**−/−**^ **Angptl7** ^**+/−**^ **and Angptl7** ^**+/+**^ **mice. (a)** Experimental design for assessing the repopulation efficiency in Angptl7-null BM environment. **(b)** Comparison of the repopulation efficiency in BM of *Angptl7* ^*−/−*^ *Angptl7* ^*+/−*^ and *Angptl7* ^*+/+*^ recipients. Data represent the means +/− s.e.m. (*n* = 8 for each group). **(c, d)** Comparison of the frequencies **(c)** and number **(d)** of LT-HSCs in BM of *Angptl7* ^*−/−*^ *Angptl7* ^*+/−*^ and *Angptl7* ^*+/+*^ recipients. Data represent the means +/− s.e.m. (*n* = 8 for each group). *P ≤ 0.05 for bar 9 versus bar 7 and bar 8. **(e)** Comparison of the second transplantation repopulation efficiency in BM of *Angptl7* ^*−/−*^ *Angptl7* ^*+/−*^ and *Angptl7* ^*+/+*^ recipients as referred in **(a)**. Data represent the means +/− s.e.m (*n* = 7 for each group). *P ≤ 0.05 for bar 6 versus bar 4 and bar 5, for bar 9 versus bar 7 and bar 8, for bar 12 versus bar 10 and bar 11. **(f)** Experimental design effect of endogenous deletion of Angptl7 in HSCs. *Angptl7* ^*−/−*^ *Angptl7* ^*+/−*^ or *Angptl7* ^*+/+*^ donor BM CD45.2 cells (5x10^5^ cells) were transplanted into lethally irradiated WT CD45.1 recipient mice. **(g)** Comparison of the repopulation efficiency of donor BM *Angptl7* ^*−/−*^ *Angptl7* ^*+/−*^ and *Angptl7* ^*+/+*^cells. Data represent the means +/− s.e.m. (*n* = 8 for each group). **(h, i)** Comparison of the frequencies **(h)** and number **(i)** of donor *Angptl7* ^*−/−*^ *Angptl7* ^*+/−*^ and *Angptl7* ^*+/+*^ LT-HSCs in BM of WT recipients. Data represent the means +/− s.e.m. (*n* = 8 for each group). **(j)** Comparison of the repopulation efficiency of *Angptl7* ^*−/−*^ *Angptl7* ^*+/−*^ and *Angptl7* ^*+/+*^ donor BM cells in second recipients mice as described in **(f)**. Data represent the means +/− s.e.m (*n* = 8 for each group).

Since Angptl7 is secreted by stromal cells and binds to HSPCs [9], we speculated that Angptl7 may play a non-cell autonomous role in reconstitution of the hematopoietic system. To evaluate the hypothesis, we compared the extent of repopulation of HSCs in *Angptl7*^−/−^, *Angptl7*^+/−^ and *Angptl7*^+/+^ recipient mice. After WT donor BM cells were injected into lethally irradiated *Angptl7*^−/−^*Angptl7*^+/−^ or *Angptl7*^+/+^ recipients without competitors (Figure 2a), we found that the reconstitution efficiencies of HSCs were similar among *Angptl7*^−/−^, *Angptl7*^+/−^ and WT recipient mice (Figure 2b), but the frequencies and numbers of LT-HSCs in *Angptl7*^−/−^ recipients were significantly lower than that in *Angptl7*^+/−^ and WT recipients, suggesting that Angptl7 supports the maintenance of the HSC pool in BM (Figure 2c-d). Continuously the BM cells from the primarily repopulated *Angptl7*^−/−^, *Angptl7*^+/−^ and WT recipient mice were collected for secondary transplantation. We found that the repopulating activity of cells originating from the primary *Angptl7*^−/−^ recipients was significantly decreased compared with those from the primary *Angptl7*^+/−^ and WT recipients at different time points (Figure 2e). To test whether Angptl7 had a cell-intrinsic effect on HSCs, we transplanted *Angptl7*^−/−^, *Angptl7*^+/−^ or WT BM cells into lethally irradiated WT recipients and measured the frequencies of donor cells in recipient mice (Figure 2f). We found the repopulation efficiencies, percentages, and numbers of donor *Angptl7*^−/−^, *Angptl7*^+/−^ and WT LT-HSCs were similar in WT recipients (Figure 2g-i). We further transplanted the BM cells from the primary recipient mice to lethally irradiated WT mice. The repopulation efficiencies of secondary transplantation were similar among *Angptl7*^−/−^, *Angptl7*^+/−^ and WT donors at indicated time points (Figure 2j). Overall, these results suggest that Angptl7 promoted HSC repopulation in a non-cell autonomous manner *in vivo*.

In this study, we demonstrated that deficiency of Angptl7 in the BM niche as a lack of exogenous Angptl7 stimulation impaired the reconstitution of HSCs in lethally irradiated mice, whereas endogenous deletion of Angptl7 in HSCs did not affect repopulation of HSCs in lethally irradiated mice. Thus, Angptl7 was indispensable for BM microenvironment to support HSC repopulation.

## Additional files

Additional file 1: Figure S1. Generation of *Angptl7* knockout mice. (A) Angptl7 is highly expressed in BM CD45-SSEA4+ cells. BM cells were collected by flow cytometry and Angptl7 expression was measured by real-time RT-PCR. All the cells sorted for analysis were gated from the CD45- fraction. The results were normalized to β-actin mRNA levels and represent the means +/- s.e.m. **P* < 0.05 versus bar 13 for bar 14. (B) Fluorescent microscopy imaging analyzes Angptl7 (green) and SEEA4 (red) cells in mouse BM cells. Nuclei were counterstained with DAPI (blue). Additional file 2: Figure S2. Treatment with Angptl7 induces the expansion of mouse HSPC. Left, Representative flow cytometric analysis of peripheral blood (PB) donor-derived CD45.1+ engraftment at 12 weeks after transplantation in mice transplanted with the progeny of 1× 10^5^ bone marrow lin- cells after 7 days culture with TSF or TSF plus 500 ng ml^−1^ Angptl7. Right, The mean levels of donor CD45.1+ cells in the PB of CD45.2+ recipient mice at 12 weeks after transplantation of CD45.1+ bone marrow Lin − cells (1 × 10^5^) or their progeny after 7 d of culture with TSF or TSF plus 500 ng ml^−1^ Angptl7. Data represent the means +/- s.e.m (*n* = 6 mice per group ). **P* < 0.05 versus bar 2 and bar 3 for bar 1, bar 2 for bar 3. Additional file 3:
**Supplemental methods.**
Additional file 4: Table S1. Primer list used in generation of *Angptl7*-deficient mice. Additional file 5: Figure S3. (a) Top, the DNA sequences of *Angptl7* locus targeted by *Angptl7*-TALENs are shown. The DNA-binding sites are in red, and the sequence between the DNA-binding sites is spacer region blue. There is a restriction site for the endonuclease DraIII between the two binding sites (blue). Bottom, schematic view of the design of TALENs targeting the *Angptl7* locus. The Angptl7 donor vector contains two homologous arms on both sides of exon 1 of *Angptl7*, a splice acceptor, encoding cDNA of Venus, and puromycin (Puro) that was driven by PGK promoter. *Angptl7*-TALEN recognition sites (purple box), SD: splice donor, SA: splice acceptor. (b) Electropherograms around the TALEN spacer in the *Angptl7* locus. The red boxes highlight the mice identified with *Angptl7* mutations. (c) DNA sequences of the *Angptl7* locus from live F0 mice identified in (b). ‘-’ represent deleted nucleotides. (d) The lack of Angptl7 in the BMs of Angptl7 knockout mice was confirmed by RT-PCR. (e) The lack of Angptl7 in the BMs of Angptl7 knockout mice was confirmed by western blots. Additional file 6: Table S2. The organ weight of *Angptl7*-deficient mice. Additional file 7: Figure S4. Relative expression levels of angiopoietin-like proteins in WT (+/+) and Angptl7-/-bone marrow stromal cells. The results were normalized to β-actin mRNA levels and represent the means +/- s.e.m. **P* < 0.05 versus bar 11 for bar 12.
