# Supplementary material for: Loss of Angiopoietin-like 7 diminishes the regeneration capacity of hematopoietic stem and progenitor cells
Source: J Hematol Oncol. 2015 Feb 6;8:7. doi: 10.1186/s13045-014-0102-4 (PMC4353465; doi:10.1186/s13045-014-0102-4)
Supplement: Additional file 1: Figure S1. — Generation of Angptl7 knockout mice. (A) Angptl7 is highly expressed in BM CD45-SSEA4+ cells. BM cells were collected by flow cytometry and Angptl7 expression was measured by real-time RT-PCR. All the cells sorted for analysis were gated from the CD45- fraction. The results were normalized to β-actin mRNA levels and represent the means +/- s.e.m. *P < 0.05 versus bar 13 for bar 14. (B) Fluorescent microscopy imaging analyzes Angptl7 (green) and SEEA4 (red) cells in mouse BM cells. Nuclei were counterstained with DAPI (blue). [file 13045_2014_102_MOESM1_ESM.doc]

**Supplementary Table 1. Primer list of Loss of Angiopoietin-like 7 diminishes the regeneration capacity of hematopoietic stem and progenitor cells.**

| **Primer Name** | **Sequence(5'to3')** | **Organism** | **Purpose** |
| --- | --- | --- | --- |
| **β-actin-F** | TTCAACACCCCAGCCATGTA | mice | RT-PCR |
| **β-actin-R** | TGTGGTACGACCAGAGGCATAC | mice | RT-PCR |
| **Angptl7-F** | TGACTGTTCTTCCCTGTACCA | mice | RT-PCR |
| **Angptl7-R** | CAAGGCCACTCTTACGTCTCT | mice | RT-PCR |
| **Angptl7 KO genotyping-F** | CTCTAGCTTTAAGAAAGGCT | mice | amplification |
| **Angptl7 KO genotyping-R** | CAGTGCTGAGCCGAGACTCC | mice | amplification |
| **Angptl7-TALEN L arm-F** | AGGGAATCGAGCCTTCTGATCTGC | mice | amplification |
| **Angptl7-TALEN L arm-R** | TggACTAGTccGAATTCggccgccctggccACGCGTcgTTCGAAggccgtaatggccACTTACCTGAACACATAGCCAGGTCTCCC | mice | amplification |
| **Angptl7-TALEN R arm-F** | gcgGAATTCACGCAAGACACAGCTCAAAGCA | mice | amplification |
| **Angptl7-TALEN R arm-R** | ggACTAGTATGACCAGTTCCATCTCACAGAAC | mice | amplification |
