# Supplementary material for: Loss of Angiopoietin-like 7 diminishes the regeneration capacity of hematopoietic stem and progenitor cells
Source: J Hematol Oncol. 2015 Feb 6;8:7. doi: 10.1186/s13045-014-0102-4 (PMC4353465; doi:10.1186/s13045-014-0102-4)
Supplement: Additional file 2: Figure S2. — Treatment with Angptl7 induces the expansion of mouse HSPC. Left, Representative flow cytometric analysis of peripheral blood (PB) donor-derived CD45.1+ engraftment at 12 weeks after transplantation in mice transplanted with the progeny of 1× 10^5^ bone marrow lin- cells after 7 days culture with TSF or TSF plus 500 ng ml−1 Angptl7. Right, The mean levels of donor CD45.1+ cells in the PB of CD45.2+ recipient mice at 12 weeks after transplantation of CD45.1+ bone marrow Lin − cells (1 × 10^5^) or their progeny after 7 d of culture with TSF or TSF plus 500 ng ml−1 Angptl7. Data represent the means +/- s.e.m (n = 6 mice per group ). *P < 0.05 versus bar 2 and bar 3 for bar 1, bar 2 for bar 3. [file 13045_2014_102_MOESM2_ESM.doc]

| **Organ(s)** | **+/+ (n = 6)** | **+/- (n=10)** | **−/− (n = 11)** |
| --- | --- | --- | --- |
| **Body weight (g)** | 27.2 ± 1.9 | 25.69 ±3.3 | 26.6 ± 2.6 |
| **Spleen weight (mg)** | 99.6 ±23.3 | 96.7 ±9.7 | 104.9 ± 27.1 |
| **Liver weight (mg)** | 1382.3 ± 264.6 | 1241.9 ±46.6 | 1283.6 ± 375.3 |
| **Kidney weight (mg)** | 301.8.0 ± 22.8 | 293.1 ±29.1 | 295.7 ± 58.1 |
| **Heart weight (mg)** | 102.7 ± 35.1 | 92.7 ±11.3 | 111.3 ± 15.9 |
| **Lung weight (mg)** | 172.6 ± 11.1 | 166.3 ±7.5 | 168.4± 17.1 |
| **Thymus weight (mg)** | 104.2 ± 5.9 | 96.9 ±12.9 | 88.6 ± 2.7* |

**Supplementary Table 2.  The weights of *Angptl7*-deficient mice were normal.**

**Supplementary Table 2.  The weights of *Angptl7*-deficient mice were normal.** Weights of whole body and different organs in wild type mice (+/+), *Angptl7* heterozygous mutant mice (+/-), and *Angptl7* homozygous mutant Mice (-/-). Body weights were shown in grams. Organ weights were shown in milligrams. Data represent the means +/- SD.
