# Supplementary figures and images for: Loss of Angiopoietin-like 7 diminishes the regeneration capacity of hematopoietic stem and progenitor cells
Source: J Hematol Oncol. 2015 Feb 6;8:7. doi: 10.1186/s13045-014-0102-4 (PMC4353465; doi:10.1186/s13045-014-0102-4)

Supplementary Figure 1

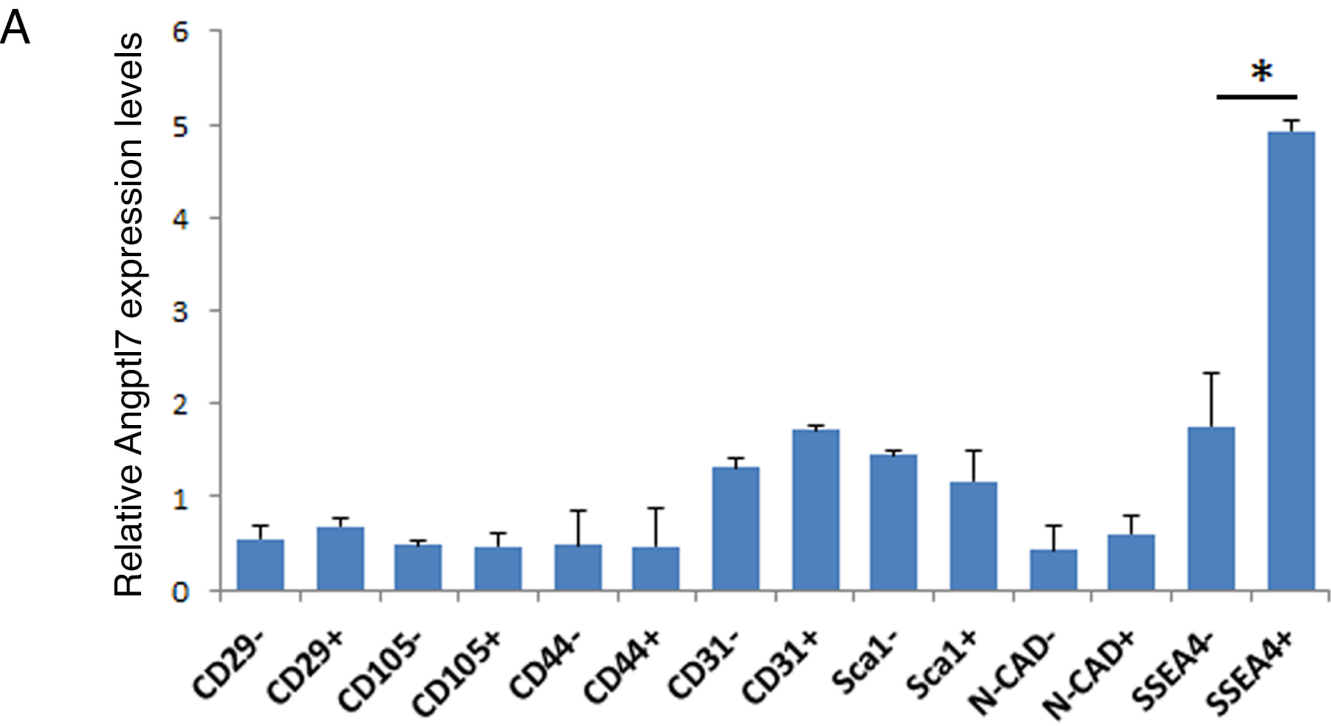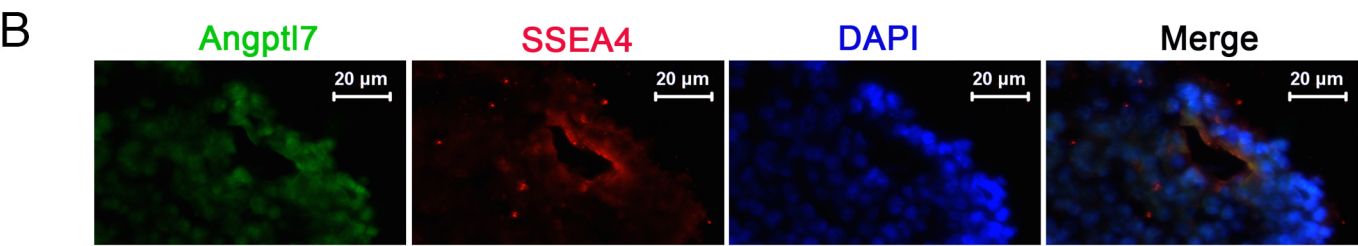

Supplement: Additional file 3: — Supplemental methods. [file 13045_2014_102_MOESM3_ESM.pdf]

Supplementary Figure 2

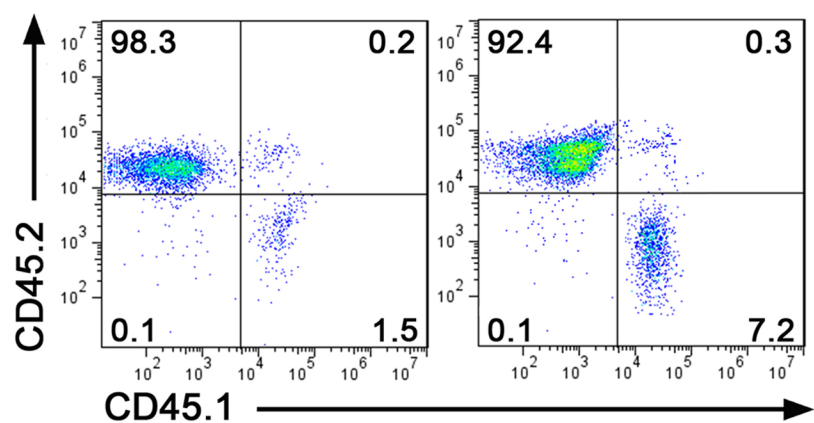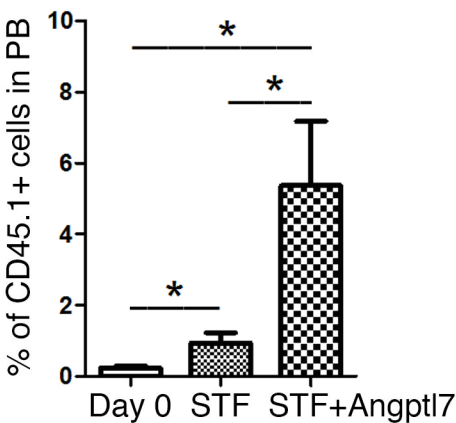

Supplement: Additional file 4: Table S1. — Primer list used in generation of Angptl7-deficient mice. [file 13045_2014_102_MOESM4_ESM.pdf]

Supplementary Figure 3

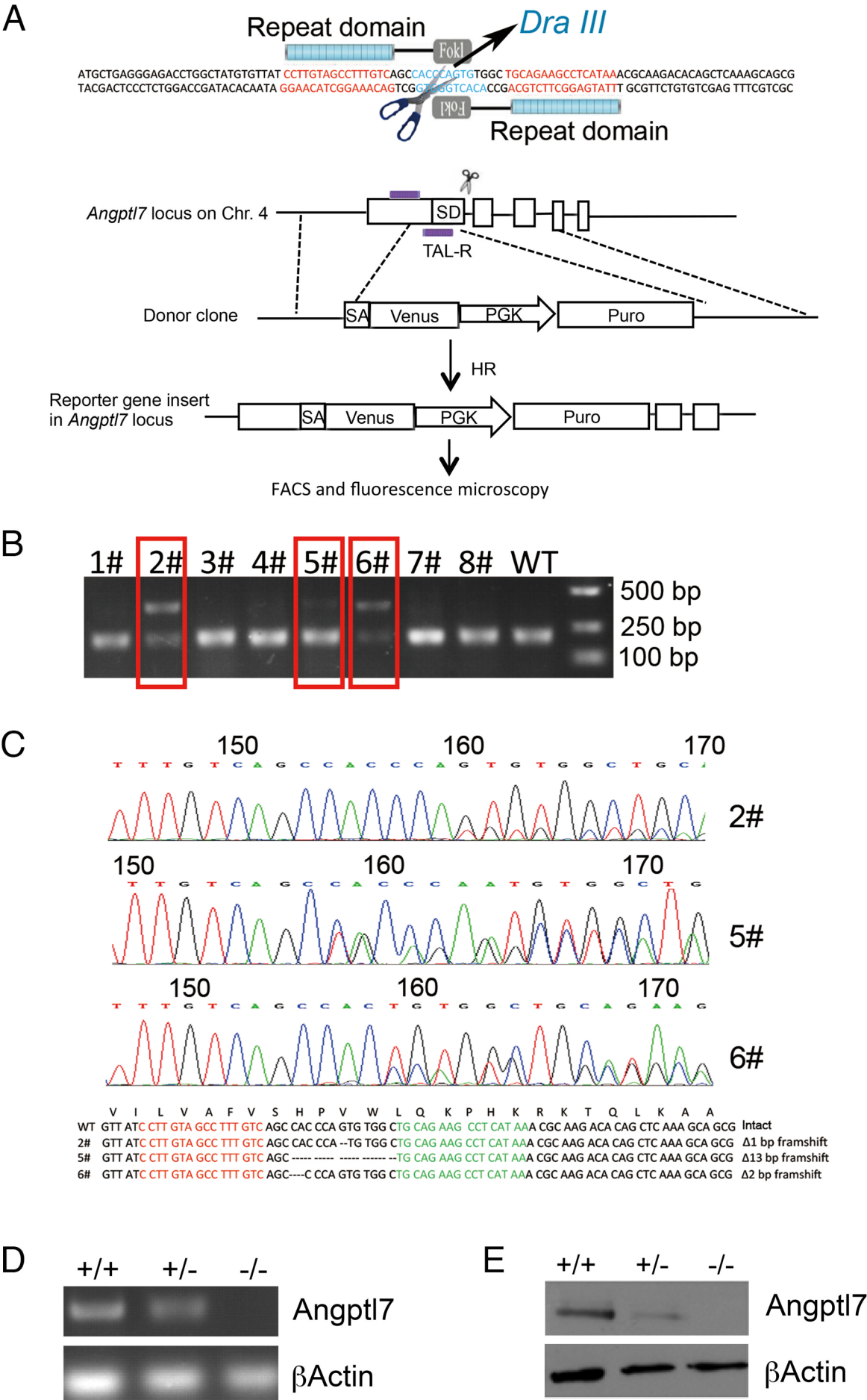

Supplement: Additional file 5: Figure S3. — (a) Top, the DNA sequences of Angptl7 locus targeted by Angptl7-TALENs are shown. The DNA-binding sites are in red, and the sequence between the DNA-binding sites is spacer region blue. There is a restriction site for the endonuclease DraIII between the two binding sites (blue). Bottom, schematic view of the design of TALENs targeting the Angptl7 locus. The Angptl7 donor vector contains two homologous arms on both sides of exon 1 of Angptl7, a splice acceptor, encoding cDNA of Venus, and puromycin (Puro) that was driven by PGK promoter. Angptl7-TALEN recognition sites (purple box), SD: splice donor, SA: splice acceptor. (b) Electropherograms around the TALEN spacer in the Angptl7 locus. The red boxes highlight the mice identified with Angptl7 mutations. (c) DNA sequences of the Angptl7 locus from live F0 mice identified in (b). ‘-’ represent deleted nucleotides. (d) The lack of Angptl7 in the BMs of Angptl7 knockout mice was confirmed by RT-PCR. (e) The lack of Angptl7 in the BMs of Angptl7 knockout mice was confirmed by western blots. [file 13045_2014_102_MOESM5_ESM.pdf]

Supplementary Figure 4

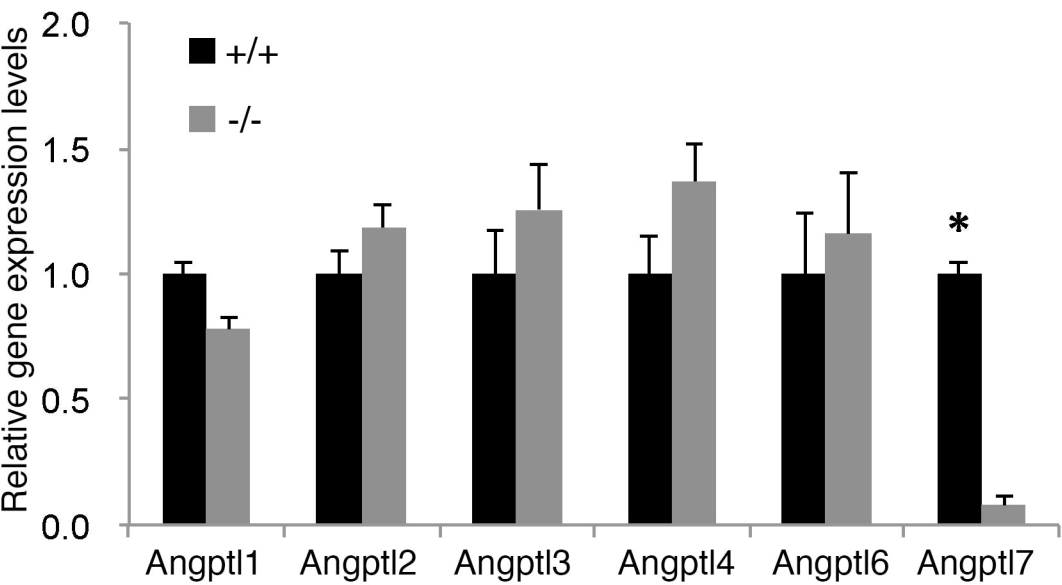

Supplement: Additional file 6: Table S2. — The organ weight of Angptl7-deficient mice. [file 13045_2014_102_MOESM6_ESM.pdf]
