# Supplementary material for: Loss of Angiopoietin-like 7 diminishes the regeneration capacity of hematopoietic stem and progenitor cells
Source: J Hematol Oncol. 2015 Feb 6;8:7. doi: 10.1186/s13045-014-0102-4 (PMC4353465; doi:10.1186/s13045-014-0102-4)
Supplement: Additional file 7: Figure S4. — Relative expression levels of angiopoietin-like proteins in WT (+/+) and Angptl7-/-bone marrow stromal cells. The results were normalized to β-actin mRNA levels and represent the means +/- s.e.m. *P < 0.05 versus bar 11 for bar 12. [file 13045_2014_102_MOESM7_ESM.docx]

**Supplementary methods**

**Cell culture**

StemSpan serum-free medium (StemCell Technologies, Canad) was used as the basal medium. The basal medium supplemented with 100 ng/mL murine SCF (Peprotech, Rochy Hill, USA), 20 ng/mL murine TPO (Peprotech, Rochy Hill, USA), and 10 ng/mL murine FLT3L (Peprotech, Rochy Hill, USA) was used as STF medium. Angptl7-6×his were purified in 3T3 cell by Ni-NTA affinity purification according to the manufacturer instructions (Qiagen, Germany). The protein concentration was determined with the Bio-Rad protein assay kit (Bio-Rad, California, USA) and BSA as the standard according to the manufacturer instructions.

**Colony-forming cell (CFC) assays**

Cell suspensions of 2×10^4^ cells/mL for mouse BM cells were used. Approximately 0.3 mL of cells was added to 3 mL of the MethoCult™ M3334 (StemCell Technologies, Canada) for duplicate cultures according to the manufacturer’s instructions. Granulocyte/macrophage CFUs and erythroid burst-forming units and CFU-E, BFU-E, CFU-GM, and CFU-GEMM were scored on day 10 of each culture.

**Flow cytometry**

Flow cytometric analysis was performed using Accuri C6 or FACSAria™ II (BD Biosciences, San Jose, CA, USA). Bone marrow cells from transplanted mice and *Angptl7*-knockout mice was stained using mouse CD45.1-PE (eBioscience, San Diego, CA, USA), mouse CD45.2-APC (eBioscience, San Diego, CA, USA), mouse CD3e-biotin (BD Pharmingen, San Jose, CA, USA), mouse CD11b-biotin (BD Pharmingen, San Jose, CA, USA), mouse B220-biotin (BD Pharmingen, San Jose, CA, USA), mouse Ly6G/C-biotin (BD Pharmingen, San Jose, CA, USA), mouse TER-119-biotin (BD Pharmingen, San Jose, CA, USA), streptavidin-FITC (eBioscience, San Diego, CA, USA), mouse c-Kit-PE (eBioscience, San Diego, CA, USA), or mouse Sca1-APC (eBioscience, San Diego, CA, USA). Mouse BM machencamal cells were sorted with mouse CD29-PE (eBioscience, San Diego, CA, USA), mouse CD44-PE (eBioscience, San Diego, CA, USA), mouse CD105-PE (eBioscience, San Diego, CA, USA), mouse CD31-PE (eBioscience, San Diego, CA, USA), mouse CD45-APC (eBioscience, San Diego, CA, USA), mouse -SSEA4 (Abcam, Cambridge, UK) and rabbit anti-mouse IgG-FITC (eBioscience, San Diego, CA, USA).

***Angptl7*-null mice genotyping**

All primers used in this study are listed in Supplementary Table 2. To genotype the *Angptl7* knockout mice, we extracted DNA from tail tips and digested in a 2 mL Eppendorf tube containing 100 mL extraction solution (50 mM Tris-HCl, pH8.0; 25 mM EDTA; and 400 mM NaCl), 100mL 10% SDS, and 20 mL Proteinase K (10 mg/mL) at 55°C overnight. After incubation, the lysates were centrifuged at 12,000 g for 15 min, and 80 mL of the supernatant was transferred into a new Eppendorf tube. Nucleic acids were precipitated by adding 800 mL cold 100% isopropyl alcohol, followed by centrifugation at 8,000 g for 10 min. Pellets were washed twice with 500 mL 70% alcohol. After drying, pellets were resuspended in 100mL TE buffer (10 mM Tris-HCl, pH 8.0; 1 mM EDTA and 50 mg/mL RNAse). DNA samples were stored at 4 °C.The Angptl7 knockout site was amplified using primers 5’–CTCTAGCTTTAAGAAAGGCT–3’ and 5’–CAGTGCTGAGCCGAGACTCC–3’. The cycling conditions were 98°C for 2 minutes; 32 cycles of 98°C for 30 seconds, 58°C for 15 seconds, and 72°C for 30 seconds; and a final extension of 72°C for 5 minutes. The amplification products were digested with *AdeI* (Thermo Scientific, Waltham, MA, USA). Western blots were performed to detect the Angptl7 protein using the rat Angptl7-specific antibody (MAB4960, R&D, Minneapolis, MN, USA) and, rat mouse β-actin-specific antibody (ab8227, Abcam, Cambridge, UK) was used as a loading control.

**TALEN and targeting-vector construction**

To design an appropriate TALEN targeting site that was convenient for identification, a targeted sequence within the first exon of Angptl7 (accession number: NM_001039554.3) was chosen. TALEN-Left (TAL-L) was designed against the sequence (5′-CCTTGTAGCCTTTGTC-3′) for the sense strand, and TALEN-right (TAL-R) was designed against the sequence (5′-TTATGAGGCTTCTGCA-3′) for the antisense strand. These two TALEN recognition sites were separated by a 16-bp spacer region (AGCCACCCAGTGTGGC). Plasmids encoding TALENs were constructed using the Golden Gate TALEN Assembly kit (Addgene) and according to the code rules as follows: HD for C, NG for T, NI for A, and NN for G. The TALENs repeat-variable diresidue domain (RVD) arrays were then sub-cloned into pCS2+ expression vectors, which contain the *FokI* nuclease.

**Competitive reconstitution analysis**

1×10^5^ mouse CD45.1 donor Lin- cells and the progeny of 1×10^5^ cultured in STF or STF plus 500 ng/ml medium for 7 days was injected intravenously via the retro-orbital route into each of a group of 6- to 9-week-old CD45.2 mice that had been previously irradiated with a total dose of 10 Gy. 1×10^5^ CD45.2 bone marrow mononuclear cells were used as competitor cells. Hematologic reconstitution engraftment in the peripheral blood was analyzed by flow cytometry over time at 12 weeks post transplantation.

**Immunofluorescence**

Freshly dissected decalcified femurs from 6- to 12-week-old WT C57BL/6 mice were embedded in OCT and chilled in dry ice for 1-2 hours. Sections were generated using Superfrost/Plus microscope slides (Fisher Scientific), Leica High Profile microtome blades (Model 818, Cat No: 63 062-01), and a Leica Cryostat with Blade Holder for a High Profile Blade at -20°C. The 8 µm sections were air-dried overnight at room temperature and subsequently fixed in -20°C methanol for 10 minutes. Slides were then blocked with 20% donkey serum in 0.1M phosphate buffer (pH 7.4) for 60 minutes and then primary Angptl7 (R&D, Minneapolis, MN, USA) and SSEA4 (Abcam, Cambridge, UK) antibodies overnight. Slides were further incubated by FITC-conjugated secondary antibody (eBioscience, San Diego, CA, USA) and Alexa Fluor 594-conjugated secondary antibody (Invitrogen, Eugene, Oregon, USA). The nuclear dye DAPI was included to evaluate nuclear morphology. Finally, slides were mounted without drying using IMMU-MOUNT (Thermo Scientific).

**Statistical analysis**

Data were analyzed using GraphPad Prism 4 with Student’s t-test. P values less than 0.05 were considered statistically significant.
